# Trends in maternal mortality in China and Hainan Province: an analysis based on GBD 2023 and Hainan surveillance data

**DOI:** 10.3389/fgwh.2026.1864385

**Published:** 2026-07-20

**Authors:** Ruili Chen, Xuejiao Li, Guihua Wu, Humin Gong

**Affiliations:** 1 Department of Obstetrics, Hainan Women and Children’s Medical Center, Haikou, Hainan, China; 2 Department of Maternal and Child Health Care and Primary Care Guidance, Hainan Women and Children’s Medical Center, Haikou, Hainan, China; 3 Hainan Medical University, Haikou, Hainan, China

**Keywords:** China, GBD 2023, Hainan Province, interrupted time series analysis, joinpoint regression, maternal mortality ratio

## Abstract

**Objective:**

To examine long-term trends in maternal mortality ratio (MMR) in China (1990–2023) and Hainan Province (2003–2023), characterize age-specific and cause-specific patterns, and assess the impact of major policy events on MMR trends.

**Methods:**

Data were obtained from the Global Burden of Disease Study 2023 and Hainan provincial maternal mortality surveillance records. Joinpoint regression was used to identify segmented trends and estimate annual percent change (APC). Log-linear regression was used to estimate the overall estimated annual percent change (EAPC). National data were used for age- and cause-specific analyses. Interrupted time series analysis assessed temporal associations of the 2009 healthcare reform, the 2016 universal two-child policy, and the 2020 COVID-19 pandemic with national MMR trends.

**Results:**

From 1990 to 2023, China's MMR decreased from 121.9 to 10.7 per 100,000 live births, a 91.3% decline, with an EAPC of −9.06% (95% CI: −9.79 to −8.32). In Hainan Province, MMR decreased from 47.5 to 8.9 per 100,000 live births during 2003–2023, an 81.4% decline, with an EAPC of −7.39% (95% CI: −9.06 to −5.72). Three national joinpoints were identified in 2004, 2013, and 2018, indicating a slow decline, rapid decline, slower decline, and stabilization during 2018–2023. No significant joinpoint was detected in Hainan Province. MMR decreased significantly across all age groups but remained relatively high among women aged 40–49 years. Mortality due to postpartum hemorrhage and hypertensive disorders of pregnancy declined markedly. Indirect maternal mortality also decreased but remained relatively high in the later period. After the 2016 policy, both the level and trend terms of MMR increased significantly. After the COVID-19 pandemic, the level term was not significant, whereas the trend term increased significantly.

**Conclusion:**

MMR declined substantially in China from 1990 to 2023 and showed a sustained decline in Hainan Province, with a markedly narrowed provincial-national gap. Older pregnant women remain a key high-risk group, and postpartum hemorrhage and hypertensive disorders of pregnancy remain major prevention priorities. After the universal two-child policy, both the level and trend terms of MMR increased significantly. After the COVID-19 pandemic, the MMR trend term also increased significantly.

## 1 Introduction

The maternal mortality ratio (MMR) is an important indicator for assessing women's health status in a country or region and a key marker of health system performance and quality of healthcare services (1). Although the global MMR has declined overall, maternal mortality remains an important cause of death among women of reproductive age in some countries and regions, with major causes including postpartum hemorrhage, hypertensive disorders of pregnancy, and sepsis (1, 2). Maternal mortality remains disproportionately high in low- and middle-income countries, posing a major global public health challenge (3). Changes in MMR are influenced by the distribution of healthcare resources, policy shifts, and public health emergencies, with substantial variation across countries and regions (4). Therefore, an in-depth analysis of temporal trends in maternal mortality and their influencing factors is of considerable importance for policy development.

Since 1990, China has achieved a substantial decline in maternal mortality through the strengthening of the maternal and child health system (5). However, urban-rural disparities, interprovincial differences, and the increasing burden of high-risk pregnancies continue to impede further reductions (6). Following the implementation of the universal two-child policy, the proportion of older pregnant women increased, which has been associated with a higher risk of high-risk pregnancies (7). In addition, the coronavirus disease 2019 (COVID-19) pandemic has had a substantial impact on maternal healthcare services (8, 9). Hainan Province offers a distinctive provincial context for examining regional progress in maternal mortality reduction in China. Beyond being China's only tropical island province, Hainan differs from many inland provinces through a combination of island geography, dependence on within-province referral networks, uneven health-resource distribution (10), ethnic and linguistic diversity, and seasonal population mobility (11). These characteristics are closely relevant to maternal health because preventable maternal deaths often depend on timely risk identification, effective referral, and access to emergency obstetric care (12). In rural and less-developed areas, relatively limited primary care capacity may affect antenatal risk screening and timely referral (13), while the concentration of Li, Miao, and other ethnic minority populations in central and southern Hainan may create heterogeneous needs for maternal health education, risk communication, and equitable service utilization (14). Seasonal population inflows may further contribute to fluctuations in local health-service demand. Therefore, Hainan provides a useful setting for evaluating regional catch-up in maternal health during China's transition toward a low-MMR stage.

Previous studies have documented the substantial decline in maternal mortality in China, together with persistent county- and province-level heterogeneity and differences in cause-specific patterns (4, 5, 15). Hainan-specific studies have also described local temporal trends in maternal mortality and predicted future changes (16, 17). However, most national or multi-provincial studies have treated Hainan mainly as one administrative unit, whereas Hainan-focused studies have largely remained descriptive and have not systematically compared Hainan with national trajectories using updated GBD 2023 estimates, local surveillance data, age- and cause-specific analyses, and policy-period trend assessment. Therefore, this study extends previous work by integrating GBD 2023 estimates for China from 1990 to 2023 with Hainan provincial surveillance data from 2003 to 2023 and applying EAPC, Joinpoint regression, and interrupted time-series analysis to evaluate Hainan as a distinctive provincial comparison setting during China's transition toward a low-MMR stage.

## 2 Methods

### 2.1 Data sources

 (1) National maternal mortality data were obtained from the GBD 2023 database (https://ghdx.healthdata.org/gbd-2023). GBD 2023, organized by the Institute for Health Metrics and Evaluation (IHME) at the University of Washington, systematically assessed the burden of 375 diseases and injuries and 88 risk factors across 204 countries and territories using the latest epidemiological evidence. Its estimates are typically reported with 95% uncertainty intervals (95% UIs), reflecting the overall uncertainty arising from data heterogeneity, model parameters, and bias correction during the modeling process (18). In this study, MMR-related estimates for China from 1990 to 2023 were extracted from the GBD 2023 results query system (18), including age-specific maternal mortality rates (women aged 15–49 years grouped into 5-year intervals) and cause-specific maternal mortality rates, all expressed per 100,000 live births.

 (2) Data for Hainan Province were obtained from surveillance records of the Health Commission of Hainan Province (http://wst.hainan.gov.cn). This surveillance system collects information on maternal deaths through a combination of routine reporting and case investigation. The main data sources include the Quarterly/Annual Maternal Mortality Surveillance Report Forms, Maternal Death Report Cards, and Maternal Death Investigation Reports (17). Annual maternal deaths and live births in Hainan Province were obtained from the provincial surveillance system. Complete and consistently defined annual data with reliable denominator information were available for 2003–2023 and were therefore used for the formal trend analysis.

In this study, all outcomes were expressed as MMR (per 100,000 live births). National data were mainly used to describe the long-term characteristics of MMR in China from 1990 to 2023 and to conduct age-specific and cause-specific analyses. Data from Hainan Province were primarily used to describe the provincial MMR trend from 2003 to 2023 and to compare it with the contemporaneous national level.

### 2.2 Data quality and potential biases

Potential biases in the data sources were considered. GBD 2023 estimates are model-based and may be influenced by the quality and availability of input data, underreporting, cause-of-death misclassification, and modeling assumptions. Hainan provincial surveillance data were derived from routine reporting and case investigations. Their completeness, diagnostic capacity, and cause-of-death attribution may have changed over time. Because maternal deaths are relatively rare events at the provincial level, annual MMR estimates in Hainan may be sensitive to small changes in the absolute number of deaths. Therefore, comparisons between national GBD estimates and Hainan surveillance data should be interpreted cautiously as descriptive comparisons rather than strictly equivalent estimates from the same data system.

Complete and consistently defined Hainan provincial maternal mortality surveillance data for 1991–2002 were not available for this study. The earlier period had missing annual observations, incomplete denominator information in some years, and limited comparability during the early standardization of the surveillance system. Because Joinpoint regression and ITSA require continuous and comparable annual time-series data, the Hainan analysis was restricted to the continuous series from 2003 to 2023. Data from 1991 to 2002 were not included in the formal trend analysis to reduce instability and potential bias caused by discontinuous observations and limited temporal comparability.

### 2.3 Descriptive analysis and EAPC estimation

Descriptive analyses were first conducted for the MMR in China and Hainan Province by comparing the MMR in the first and last study years and calculating the overall magnitude of decline. Annual trend plots were generated for MMR by age group and cause of death to visually illustrate long-term changes.

The estimated annual percent change (EAPC) is a commonly used indicator of overall trend in recent GBD-related studies and analyses of maternal disease burden (19). In this study, EAPC was used to evaluate the overall trend in MMR during the study period. A log-linear regression model was fitted with annual MMR as the dependent variable and calendar year as the independent variable:

$$\ln\;(\mathrm{MM}R_{t})=\alpha +\beta t+\varepsilon_{t}$$

where MMR_t_ represents the maternal mortality ratio in year t, ln(MMR_t_) represents its natural logarithm, t is calendar year, α is the intercept, β is the regression coefficient, and ε_t_ is the random error term. EAPC was calculated as follows:

EAPC=100×[exp(β)−1]

with corresponding 95% confidence intervals (95% CIs). If the EAPC and the upper bound of its 95% CI were both below 0, the MMR was considered to show a decreasing trend. If both the EAPC and the lower bound of its 95% CI were above 0, an increasing trend was considered present. If the 95% CI crossed 0, the trend was considered statistically nonsignificant.

### 2.4 Joinpoint regression analysis

Joinpoint regression has been used to analyze long-term trends in maternal and child health indicators (20). In this study, Joinpoint regression was applied to assess temporal trends and segmented characteristics of MMR in China and Hainan Province. The Joinpoint regression model was specified as a segmented log-linear regression (21):

$$\ln\;(\mathrm{MM}R_{t})=\beta_{0}+\beta_{1}t+\overset{K}{\underset{k=1}{\sum }}\;\delta_{k}(t-\tau_{k}{)}_{+}+\;\varepsilon_{t}$$

where MMR_t_ represents the maternal mortality ratio in year t, and ln(MMR_t_) represents its natural logarithm; t is calendar year; K is the total number of joinpoints; τ_k_ denotes the kth joinpoint; and (t−τ_k_)_+_ = max(0,t−τ_k_), which equals 0 before the corresponding joinpoint and equals t−τ_k_ thereafter. β_0_ is the intercept, β_1_ is the initial slope, δ_k_ denotes the change in slope after the kth joinpoint, and *ε*_t_ is the random error term. Natural log transformation of MMR was used to account for heteroscedasticity, and permutation tests were applied to determine the optimal number of joinpoints.

A Joinpoint regression model was fitted using calendar year as the independent variable and the natural logarithm of MMR as the dependent variable. By identifying turning points in the time series, the overall trajectory was partitioned into several linear segments, and the annual percent change (APC) and its 95% CI were calculated for each segment to characterize stage-specific trends. The analysis period was 1990–2023 for national data and 2003–2023 for Hainan Province. The number of joinpoints was set between 0 and 3. Permutation tests were used to identify the optimal model, and the number of joinpoints, segment-specific APCs, 95% CIs, and *P* values were reported. An APC was considered statistically significant when its 95% CI did not include 0. Long-term trends in age-specific MMR were evaluated using EAPC and its 95% CI to assess the direction and statistical significance of changes across age groups.

### 2.5 Interrupted time series analysis

ITSA is a commonly used quasi-experimental approach for evaluating public health policies and COVID-19-related changes in health outcomes in China (22). To assess the temporal associations between major policies/public health events and changes in MMR, ITSA was conducted using the national MMR time series from 1990 to 2023. Based on the study objectives, three intervention time points were prespecified: the launch of the 2009 healthcare reform, the implementation of the universal two-child policy in 2016, and the onset of the COVID-19 pandemic in 2020. A segmented log-linear regression model incorporating these three intervention points was fitted to estimate the baseline trend before the first intervention and the changes in level and slope associated with each policy or public health event. The level-change coefficients represent the immediate change in MMR after each intervention. The slope-change coefficients represent the difference between the post-intervention trend and the pre-intervention trend, rather than the absolute slope of that period. The general form of the model was:

$$\ln\;(\mathrm{MM}R_{t})=\beta_{0}+\beta_{1}\mathrm{Tim}e_{t}+\beta_{2}I_{2009,t}+\beta_{3}\times \mathrm{Post}2009_{t}+\beta_{4}I_{2016,t}+\beta_{5}\times \mathrm{Post}2016_{t}+\beta_{6}I_{2020,t}+\beta_{7}\times \mathrm{Post}2020_{t}+\varepsilon_{t}$$

where MMR_t_ represents the maternal mortality ratio in year t; Time_t_ is the continuous time variable; I_2009,t_, I_2016,t_, and I_2020_,_t_ are indicator variables for the post-intervention periods of 2009, 2016, and 2020, respectively (coded as 0 before the intervention and 1 in the intervention year and thereafter); Post2009_t_, Post2016_t_, and Post2020_t_ are continuous time variables after each intervention point (coded as 0 before and during the intervention year, 1 in the first year after the intervention, and increased by 1 in each subsequent year); and ε_t_ is the random error term. In this model, β_0_ represents the baseline level, and β_1_ represents the baseline slope before the first intervention. The coefficients β2, β4, and β6 represent immediate level changes after the 2009, 2016, and 2020 intervention points, respectively. The coefficients β3, β5, and β7 represent changes in slope after each intervention relative to the preceding trend. Level-change and slope-change coefficients were transformed using 100 × [exp(β)−1]. Level changes are reported as percentage changes, and slope changes are reported as percentage changes per year.

The analysis was intended to assess temporal associations rather than causal effects. Candidate segmented models allowing 0 to 4 interruption points were compared using the Bayesian information criterion (BIC), and the model with the lowest BIC was selected as the best-fitting model.

### 2.6 Statistical analysis

All statistical tests were two-sided, and a *P* value <0.05 was considered statistically significant. Original estimates extracted from the GBD database were presented with 95% UIs, whereas EAPC, APC, and ITSA regression coefficients were reported with 95% CIs to reflect the precision and statistical significance of point estimates. Descriptive analyses were used to present the long-term changes in MMR at the national and provincial levels, as well as across age groups and causes of death. EAPC was used to evaluate the overall trend. Joinpoint regression was used to identify turning points and segmented changes. ITSA was used only to assess level and trend changes in national MMR after major policy and event nodes. All data management, statistical analyses, and figure generation were conducted in R version 4.3.1, and Joinpoint regression was performed using Joinpoint Regression Program version 4.9.1.0.

## 3 Results

### 3.1 Temporal trends and segmented changes in maternal mortality in China and Hainan Province

**Figure 1:**
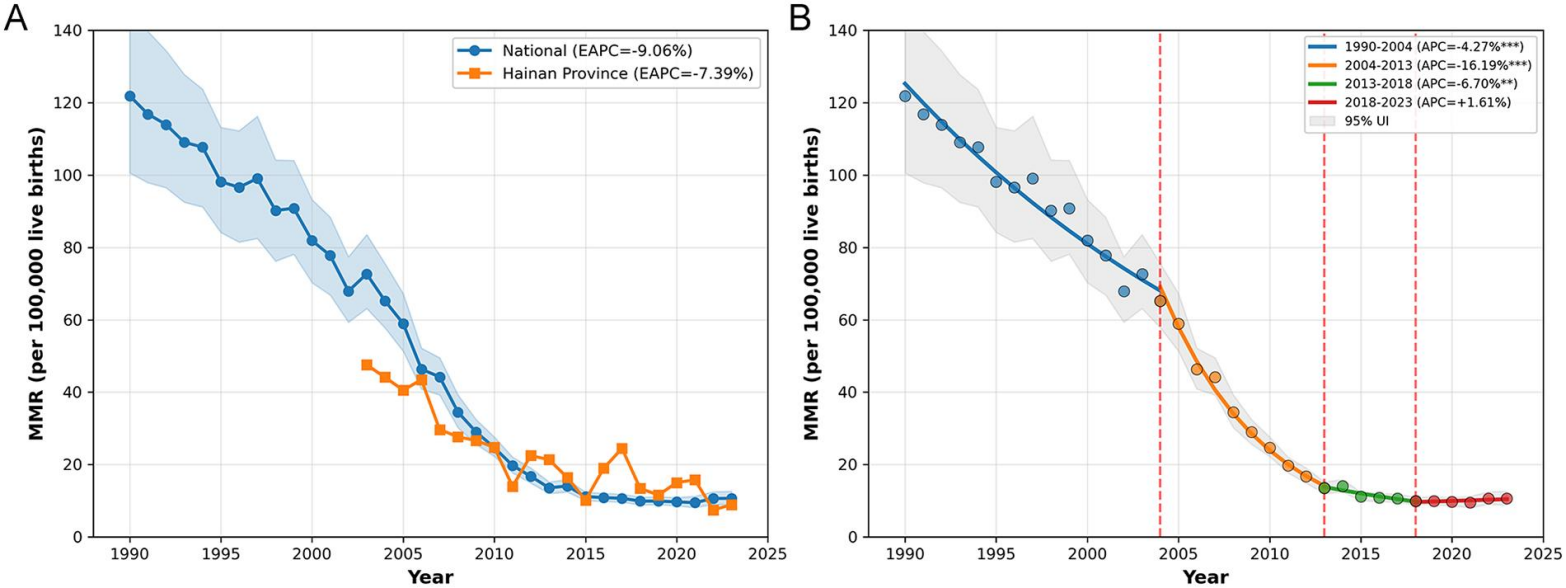
Temporal trends in maternal mortality ratio (MMR) in China and Hainan Province and joinpoint regression analysis. **(A)** Comparison of MMR trends between China and Hainan Province, 2003–2023. **(B)** Joinpoint regression fit of national MMR in China, 1990–2023. Red dashed lines indicate identified joinpoints, and solid lines represent fitted trends for each segment. EAPC, estimated annual percent change; APC, annual percent change; UI, uncertainty interval. **P < 0.01 and ***P < 0.001 indicate statistical significance.

From 1990 to 2023, the national MMR in China declined from 121.9 to 10.7 per 100,000 live births. This represented an overall reduction of 91.3%, with an EAPC of −9.06% (95% CI: −9.79 to −8.32). From 2003 to 2023, the MMR in Hainan Province declined from 47.5 to 8.9 per 100,000 live births, corresponding to an 81.4% reduction, with an EAPC of −7.39% (95% CI: −9.06 to −5.72) (Figure 1A, Table 1). Joinpoint regression identified three joinpoints in the national MMR trajectory, whereas no significant joinpoint was identified for Hainan Province, suggesting a continuous downward trend throughout the observation period (Table 1). Further segmented analysis of the national series showed that MMR declined slowly during 1990–2004 (APC = −4.27%, 95% CI: −4.74 to −3.79; *P* < 0.001), declined more rapidly during 2004–2013 (APC = −16.19%, 95% CI: −16.99 to −15.39; *P* < 0.001), and then showed a moderated decline during 2013–2018 (APC = −6.70%, 95% CI: −9.39 to −4.02; *P* = 0.009). During 2018–2023, the trend was no longer statistically significant (APC = 1.61%, 95% CI: −0.43 to 3.65; *P* = 0.194) (Figure 1B, Table 2). During the overlapping observation period from 2003 to 2023, the gap in MMR between the national level and Hainan Province narrowed from 18.5 to 1.8 per 100,000 live births.

**Table 1:** Overall indicators of maternal mortality ratio (MMR) in China and Hainan Province.

Indicator	China	Hainan province
Study period	1990–2023	2003–2023
Initial MMR (per 100,000 live births)	121.9	47.5
Final MMR (per 100,000 live births)	10.7	8.9
Percentage decline (%)	91.3	81.4
EAPC (%)	−9.06	−7.39
95% CI	[−9.79, −8.32]	[−9.06, −5.72]
Number of joinpoints	3	0

EAPC, estimated annual percent change; CI, confidence interval.

**Table 2:** Joinpoint regression analysis of maternal mortality ratio (MMR) in China, 1990–2023.

Period	APC (%)	95% CI	*P* value
1990–2004	−4.27	[−4.74, −3.79]	<0.001
2004–2013	−16.19	[−16.99, −15.39]	<0.001
2013–2018	−6.7	[−9.39, −4.02]	0.009
2018–2023	1.61	[−0.43, 3.65]	0.194

APC, annual percent change; CI, confidence interval.

### 3.2 Temporal patterns of maternal mortality across age groups

**Figure 2:**
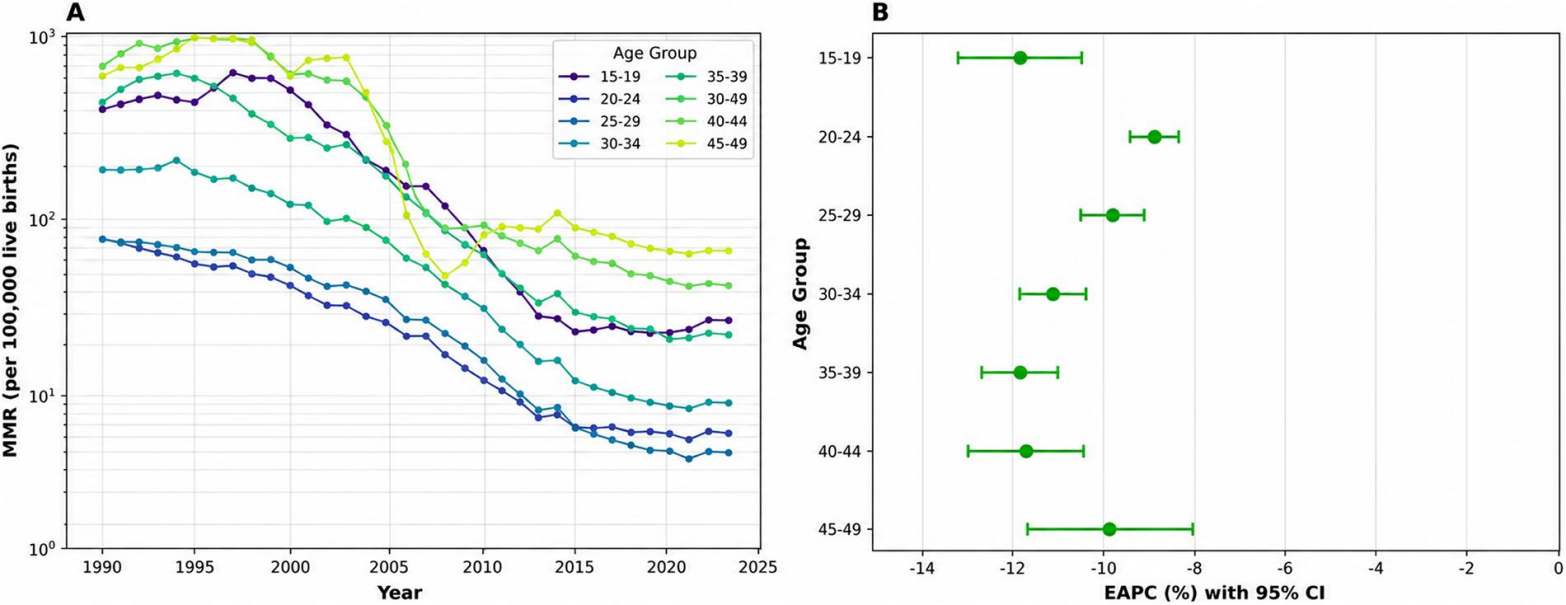
Trends in maternal mortality ratio (MMR) across age groups and corresponding estimated annual percent change (EAPC), 1990–2023. **(A)** Temporal trends in MMR across age groups (women aged 15–49 years in 5-year intervals). The *y*-axis is presented on a logarithmic scale. **(B)** Forest plot of EAPC for MMR across age groups. Error bars indicate 95% confidence intervals. EAPC, estimated annual percent change; CI, confidence interval. A trend was considered statistically significant when the 95% CI did not include 0.

From 1990 to 2023, MMR declined continuously across all age groups (Figure 2A). Throughout the study period, women aged 40–44 years and 45–49 years consistently had relatively high MMRs. In contrast, women aged 20–24 years and 25–29 years maintained relatively low levels. By 2023, MMR had decreased substantially in all age groups compared with 1990, with the lowest levels observed in the 20–24-year and 25–29-year groups. EAPC analysis showed negative EAPC values across all age groups. The upper bounds of all corresponding 95% CIs were below 0, indicating statistically significant downward trends (Figure 2B). Among these groups, the largest declines were observed in the 15–19-year and 35–39-year groups, whereas the smallest decline was observed in the 20–24-year group.

### 3.3 Temporal trends in cause-specific maternal mortality

**Figure 3:**
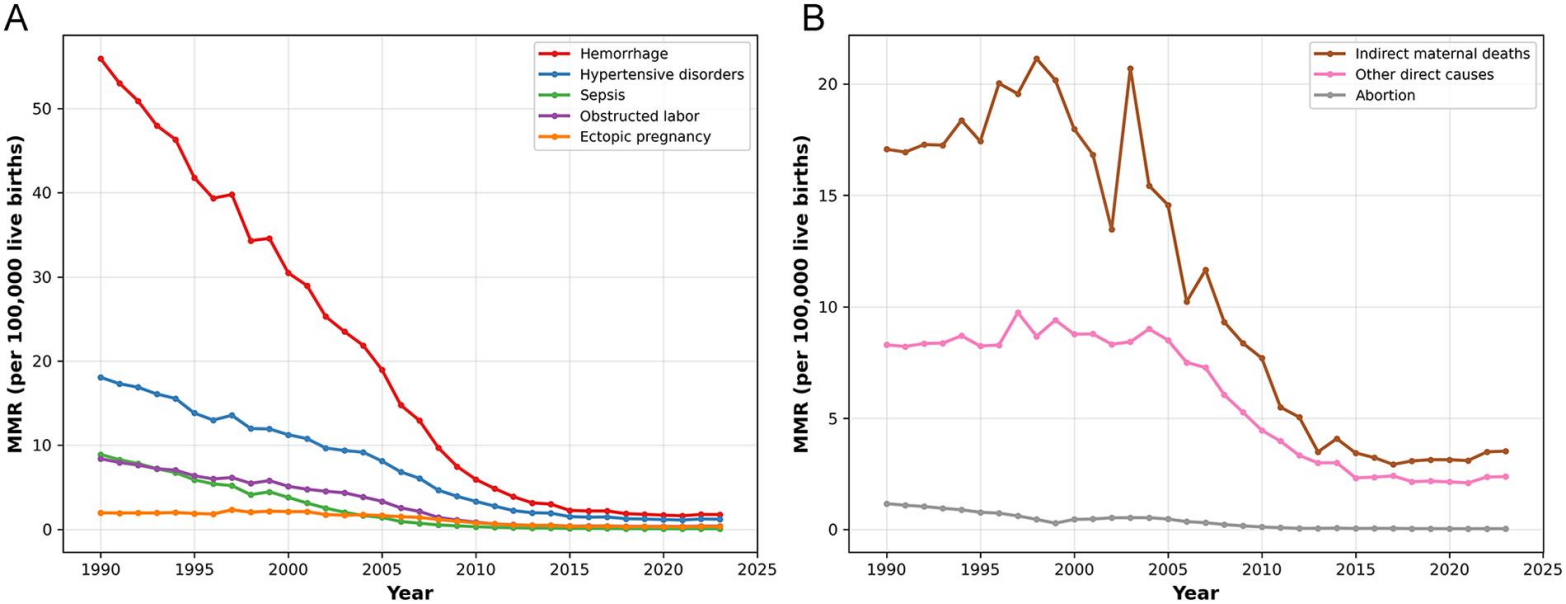
Temporal trends in cause-specific maternal mortality ratio (MMR), 1990–2023. **(A)** Direct obstetric causes, including postpartum hemorrhage, hypertensive disorders of pregnancy, sepsis, obstructed labor, and ectopic pregnancy. **(B)** Indirect and other causes, including indirect causes, other direct causes, and abortion-related mortality.

From 1990 to 2023, maternal mortality attributable to different causes showed an overall downward trend. Among direct obstetric causes, postpartum hemorrhage remained the leading cause throughout the study period. Mortality due to postpartum hemorrhage declined from approximately 56 per 100,000 live births in 1990 to approximately 1.7 per 100,000 live births in 2023. Mortality due to hypertensive disorders of pregnancy also decreased markedly, from approximately 17.8 to approximately 0.7 per 100,000 live births. In the early study period, mortality due to sepsis and obstructed labor was approximately 8.8 and 8.5 per 100,000 live births, respectively, both declining to approximately 0.2 per 100,000 live births by 2023. Mortality due to ectopic pregnancy remained relatively stable at 2.0–2.5 per 100,000 live births between 1990 and 2003, and then gradually declined to approximately 0.1 per 100,000 live births in 2023 (Figure 3A). Among indirect and other causes, mortality due to indirect causes fluctuated from the 1990s to the mid-2000s, with relatively high values around 1998 and 2003, before gradually declining after 2005 to approximately 3.5 per 100,000 live births in 2023. Mortality due to other direct causes remained at 8–10 per 100,000 live births between 1990 and 2005 and then declined to approximately 2.3 per 100,000 live births. Abortion-related mortality remained at a relatively low level throughout the study period, declining gradually from approximately 1.1 per 100,000 live births in 1990 to nearly zero after 2015, and remaining extremely low thereafter (Figure 3B).

### 3.4 Time-series results of major policy and public health event interventions on maternal mortality

**Figure 4:**
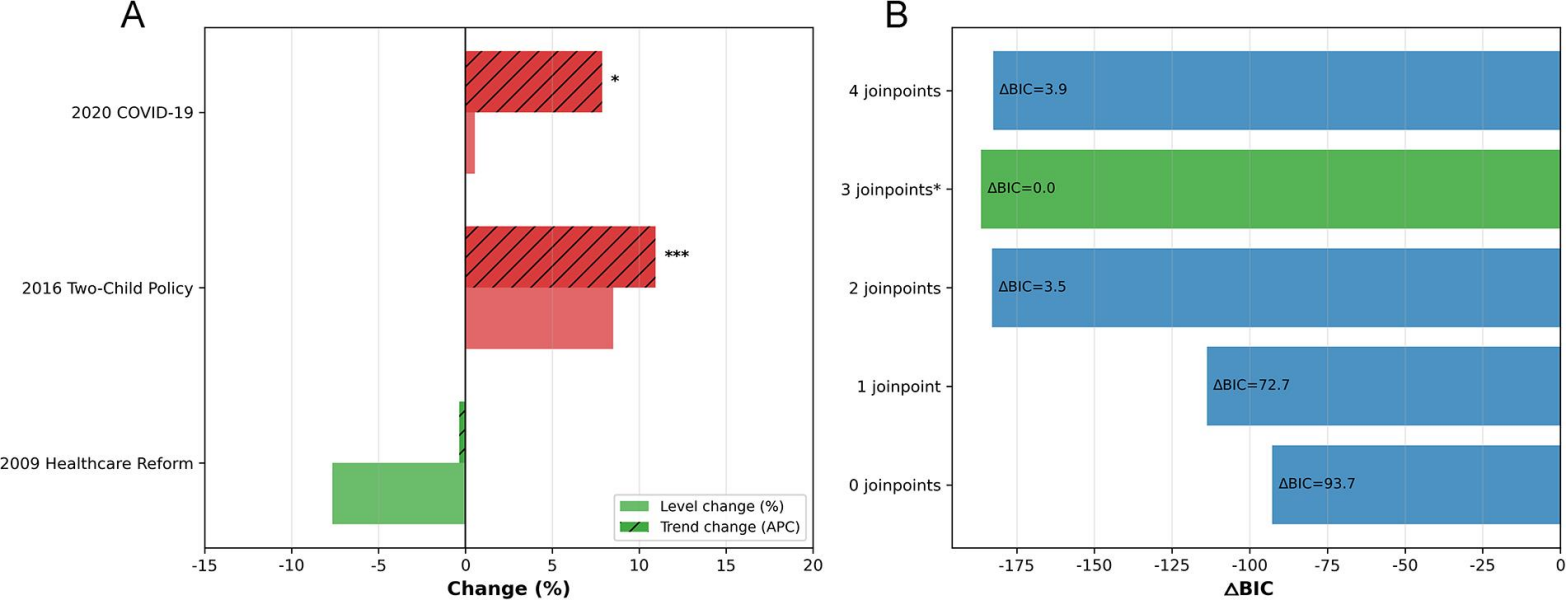
Interrupted time series analysis (ITSA) of major policy and event interventions on maternal mortality ratio (MMR) and model comparison. **(A)** Estimated level changes and slope changes in MMR following the 2009 healthcare reform, the implementation of the universal two-child policy in 2016, and the onset of the COVID-19 pandemic in 2020. **(B)** Comparison of Bayesian information criterion (BIC) values across candidate models with different numbers of interruption points. The *x*-axis represents ΔBIC, and the *y*-axis represents candidate models. ITSA, interrupted time series analysis; BIC, Bayesian information criterion; *Δ*BIC indicates the difference between the BIC of each candidate model and that of the best-fitting model. *P < 0.05, ***P < 0.001, indicating statistical significance.

Interrupted time series analysis showed that after the launch of the 2009 healthcare reform, the immediate level change in MMR was −7.67%, and the slope change was −0.35% per year; neither change reached statistical significance (Figure 4A, Table 3). Following the implementation of the universal two-child policy in 2016, the level of MMR increased significantly by 8.51%, and the slope change also increased significantly by 10.94% per year, suggesting a slowing of the previous downward trend (Figure 4A, Table 3). After the onset of the COVID-19 pandemic in 2020, the immediate level change in MMR was 0.55%, but this change was not statistically significant. However, the slope change increased significantly by 7.86% per year, suggesting a further slowing of the previous downward trend in MMR (Figure 4A, Table 3). Because only four annual observations were available after 2020, the post-COVID slope-change estimate should be interpreted with caution. Model comparison showed that, among candidate models allowing 0 to 4 interruption points, the model with three interruption points had the lowest BIC value (ΔBIC = 0.0), indicating the best fit (Figure 4B).

**Table 3:** Effects of major policy and event interventions on maternal mortality ratio (MMR) based on interrupted time series analysis.

Intervention year	Policy/event	Level change (%)	Slope change (% per year)
2009	Launch of healthcare reform	−7.67	−0.35
2016	Universal two-child policy	8.51**	10.94***
2020	COVID-19 pandemic	0.55	7.86*

* *P* < 0.05.

** *P* < 0.01.

*** *P* < 0.001, indicating statistical significance.

## 4 Discussion

This study showed a substantial decline in MMR in China from 1990 to 2023. Hainan Province showed a similar downward trend from 2003 to 2023, and the gap between Hainan and the national level narrowed markedly. These findings are consistent with previous studies showing long-term improvements in maternal and child health indicators in China, including reductions in maternal mortality and improvements in maternal healthcare services (20, 23). The sustained national decline likely reflects the combined effects of strengthened maternal and child health systems, improved antenatal care coverage, increased institutional delivery, and better management of obstetric emergencies. It may also be related to the development of referral and treatment networks for critically ill pregnant women. However, the decline in maternal mortality was not uniform over time. The trend was characterized by an initial slow decline, followed by a more rapid decline and subsequent stabilization. This pattern is consistent with findings from Shenzhen, Jinan, and other regions, which suggest that although maternal mortality has improved overall, the pace of decline has slowed in recent years (15, 24, 25). This stabilization may indicate that China has entered a low-MMR stage, in which further reductions are more difficult to achieve. At this stage, easily preventable direct obstetric deaths have been substantially reduced, and remaining deaths may be increasingly related to high-risk pregnancies, comorbidities, delayed referral, and uneven regional service capacity (26, 27).

Although MMR in Hainan Province also declined, the rate of decline was slightly slower than that at the national level, and no significant joinpoint was observed. This may indicate that improvement in Hainan was gradual rather than abrupt. It may also partly reflect the relatively small number of maternal deaths at the provincial level, which can reduce the statistical power to detect discrete turning points. Previous studies have reported a sustained decline in maternal mortality in Hainan Province, but regional differences in healthcare resources and service capacity may still influence the pace of improvement (16, 17). The narrowing gap between Hainan and the national level indicates important progress in provincial maternal health management, although the magnitude of decline remained somewhat smaller. This finding is consistent with previous evidence showing that interprovincial disparities, socioeconomic development, and healthcare accessibility influence MMR (28–30). However, the narrowing gap should not be interpreted as evidence that regional disparities have been fully resolved. Instead, it suggests that national averages may mask local differences in healthcare access, referral capacity, emergency obstetric care, and high-risk pregnancy management. Therefore, further reduction of maternal mortality in Hainan requires not only maintaining broad maternal healthcare coverage, but also strengthening county-level risk identification, emergency referral pathways, and multidisciplinary management for high-risk pregnant women.

In the age-stratified analysis, MMR declined across all age groups. However, it remained relatively high among women aged 40–49 years and relatively low among those aged 20–29 years, consistent with the important role of maternal age in maternal mortality (31). The elevated risk of maternal death among older pregnant women may be related to the increased prevalence of pregnancy complications and the rising proportion of advanced maternal age pregnancies after the implementation of the two-child policy (32–34). In the present study, the relatively large decline in MMR among women aged 35–39 years suggests that high-risk screening, hierarchical management, and early warning systems may have achieved some success (35, 36). By contrast, the smaller decline in the 20–24-year group may reflect a lower baseline risk and less room for further reduction, rather than insufficient prevention and control (25). Future efforts to reduce maternal mortality should therefore focus more on the precise identification, continuous monitoring, and multidisciplinary management of older pregnant women and those with comorbidities.

With respect to cause-specific patterns, postpartum hemorrhage remained at a relatively high level, although mortality from this cause declined substantially. This finding suggests that standardized prevention, protocol-based management, and systematic emergency care have played a key role in reducing preventable direct obstetric deaths, consistent with previous studies from mainland China (37–39). The declines in hypertensive disorders of pregnancy and sepsis are also likely related to improvements in perinatal surveillance and the management of critically ill pregnant women. However, among older women and those with comorbidities, these conditions may still contribute to the residual burden of maternal mortality (40–42). In addition, the decline in mortality due to ectopic pregnancy may be associated with advances in early diagnosis and minimally invasive treatment (43, 44). In contrast, the relatively slower decline in mortality due to indirect causes suggests an increasing need to address comorbidity-related maternal deaths. Indirect maternal deaths are commonly related to pre-existing diseases or diseases that develop during pregnancy and are aggravated by the physiological effects of pregnancy (45). Potential contributors include chronic hypertension, cardiovascular disease, diabetes, obesity, renal disease, liver disease, and other metabolic or systemic disorders, particularly among women of advanced maternal age (46). Delayed recognition of non-obstetric complications, weak multidisciplinary coordination, and unequal access to tertiary care may further contribute to preventable indirect deaths (47). Therefore, future maternal health strategies in China should place greater emphasis on preconception risk assessment, early identification of chronic diseases, multidisciplinary management, and integrated referral pathways for pregnant women with comorbidities.

Regarding policy and public health event nodes, no significant immediate change was observed following the 2009 healthcare reform. This suggests that the effects of health system reform may be cumulative and long-term rather than immediate. This interpretation is consistent with the gradual manifestation of public health policy effects (20, 22). The increase in MMR after the 2016 policy node may be related to changes in the proportion of older pregnant women and high-risk pregnancies (7, 23). After the onset of the COVID-19 pandemic in 2020, no significant level change in MMR was observed. However, the positive slope change suggests that the pandemic may have affected maternal outcomes. Potential mechanisms include reduced healthcare accessibility and delayed management of high-risk pregnancies. This finding is consistent with previous domestic and international studies (8, 9). Overall, this study provides a comprehensive analysis of changes in maternal mortality in China and offers evidence to support a shift in future prevention priorities, particularly in the management of older women, high-risk pregnancies, indirect causes of death, and the strengthening of regional referral and treatment capacity.

This study has several limitations. First, this study was based on aggregated data. Individual-level information on underlying diseases, parity, socioeconomic status, and health service utilization was unavailable. Therefore, potential confounding could not be fully controlled, and strict causal inference was limited. Second, although GBD estimates have good long-term comparability, they may still be affected by the quality of original data, underreporting, misclassification, and model estimation error. Third, the observation period for Hainan Province was shorter than that for the national data, which may affect direct long-term comparisons between the two. Finally, ITSA reflects temporal associations rather than causal effects. The results may still be influenced by concurrent policies, improvements in healthcare services, and changes in population fertility structure; therefore, the interpretation of policy- and event-related effects should remain cautious.

Future research should integrate individual-level clinical, obstetric, and health service utilization data to clarify the mechanisms underlying residual maternal mortality. County-level studies in Hainan Province are needed to identify local disparities in healthcare access, referral capacity, and emergency obstetric care. Further research should also focus on indirect maternal deaths, especially those related to cardiovascular, metabolic, and chronic diseases among women of advanced maternal age. Future policy evaluations should account for concurrent health system reforms, fertility structure changes, and regional differences in service capacity.

In conclusion, from 1990 to 2023, MMR in China declined substantially overall, and Hainan Province also demonstrated a sustained downward trend, with a markedly narrowed gap relative to the national level. Nevertheless, older pregnant women remain a key high-risk population, and postpartum hemorrhage and hypertensive disorders of pregnancy continue to be major priorities for prevention and control. MMR showed a significant upward level and trend change after the implementation of the universal two-child policy, and the trend term also increased after the COVID-19 pandemic.

## Data Availability

The original contributions presented in the study are included in the article. Further inquiries can be directed to the corresponding author.
